# The Myosin Va Head Domain Binds to the Neurofilament-L Rod and Modulates Endoplasmic Reticulum (ER) Content and Distribution within Axons

**DOI:** 10.1371/journal.pone.0017087

**Published:** 2011-02-16

**Authors:** Mala V. Rao, Panaiyur S. Mohan, Asok Kumar, Aidong Yuan, Lee Montagna, Jabbar Campbell, Veeranna, Enilza M. Espreafico, Jean P. Julien, Ralph A. Nixon

**Affiliations:** 1 Nathan Kline Institute, New York University School of Medicine, Orangeburg, New York, United States of America; 2 Department of Psychiatry, New York University School of Medicine, Orangeburg, New York, United States of America; 3 Department of Cell Biology, New York University School of Medicine, Orangeburg, New York, United States of America; 4 Departments of Cellular and Molecular Biology and Pathogenic Bioagents, Ribeirão Preto, São Paulo, Brazil; 5 Laboratory of Molecular Endocrinology, Centre de Recherche du Centre Hospitalier de l'Université Laval, Department of Anatomy and Physiology, Laval University, Québec City, Québec, Canada; Biological Research Center of the Hungarian Academy of Sciences, Hungary

## Abstract

The neurofilament light subunit (NF-L) binds to myosin Va (Myo Va) in neurons but the sites of interaction and functional significance are not clear. We show by deletion analysis that motor domain of Myo Va binds to the NF-L rod domain that forms the NF backbone. Loss of NF-L and Myo Va binding from axons significantly reduces the axonal content of ER, and redistributes ER to the periphery of axon. Our data are consistent with a novel function for NFs as a scaffold in axons for maintaining the content and proper distribution of vesicular organelles, mediated in part by Myo Va. Based on observations that the Myo Va motor domain binds to intermediate filament (IF) proteins of several classes, Myo Va interactions with IFs may serve similar roles in organizing organelle topography in different cell types.

## Introduction

Cellular transport is mediated by molecular motor proteins including kinesin, dynein/dynactin complex, and myosins. Kinesin and dynein/dynactin motors are powered by microtubule-dependent mechanisms, whereas myosin motor proteins move their cargoes by a “hand-over hand” mechanism along actin filaments [1]. The major proposed cargoes of Myo Va are membranous organelles, including melanosomes, synaptic vesicles, endosomes and mitochondria [2], [3]. Within the super family of myosin motors, myosin V is highly enriched in brain, and exists as 3 different isoforms in vertebrates: Myo Va, Vb and Vc [4]. Myo Va, which is highly conserved from yeast to mammals [5], [6], is composed of an amino terminal head domain containing an ATPase and an actin binding domain, a small neck containing calmodulin-binding IQ motifs, and a tail containing coiled-coil dimerization domains interrupted by noncoiled-coil regions and a globular domain involved in cargo binding [7], [8], [9]. The Myo Va motor complex includes two heavy chains, 12 calmodulins that bind to the neck region, and a dynein light chain 2 [10], and calmodulin kinase II, both of which bind to the tail region [11], [12].

Myo Va in neurons is believed to transport synaptic vesicles, ER, mitochondria and membrane bound vesicles along axons and within synaptic terminals, and to facilitate the accumulation of mRNA/protein complexes in dendritic spines [13]. Its special importance in the nervous system is suggested by the fact that Myo Va mutations cause a neurodevelopmental disorder, Griscelli Syndrome type 1, which is characterized by mental retardation, seizures and death early in life [14]. Myo Va mutations in mice cause an analogous syndrome, the *dilute-lethal* [dl] phenotype [6].

More recently, Myo Va has been shown to bind to neurofilaments (NFs), and other intermediate filament (IF) proteins from different cell types [15], [16]. NFs in the CNS are assembled from four subunits, the neurofilament light (NF-L), middle (NF-M), heavy (NF-H) subunits, and α-internexin [17]. NF networks are extensively cross-linked with actin filaments and microtubules [18], [19]. Myo Va binds to the NF-L subunit of NFs, which is essential for maintaining normal Myo Va levels while, loss of Myo Va leads to altered NF organization in axons [16]. However, the biological significance of the binding between Myo Va and NF-L is not clear.

In this study, we demonstrate that NF-L, rod domain, binds directly the N-terminal motor domain of Myo Va. It is well-established that the Myo Va cargo domain binds vesicular organelles (7–9), and our morphological and fractionation data (this study) demonstrate association among vesicular organelles, Myo Va, and NF-L. We showed that loss of NF-L and Myo Va leads to reduction in axonal levels of organelle markers and increased peripheral distribution of ER toward the actin-rich subaxolemmal region. Collectively, our studies provide evidence that Myo Va binding to NF-L modulates the distribution of vesicular organelles in axons. The binding of various IFs to the Myo Va head domain raises the possibility that IFs may facilitate Myo Va-mediated distribution of organelles along multiple IF systems in different cell types.

## Methods

### Ethics Statement

All the animal protocols described in the paper were approved by the Nathan Kline Institute Animal Care and Use Committee Protocol AP2005-155.

### Mutant mice

NF-L null mice are provided by Dr. Jean-Pierre Julien, Laval University, Canada [20]; Myo Va mutants (DL20J breeders [21]) were fed with high fat (9%) diet (Purina, St. Louis, MO), and are a kind gift of Dr. Nancy A. Jenkins (NCI, MD). NF-L null mice are in mixed 129/BL6/J while DL20J are in pure BL6/J genetic background. Animals are maintained at 12 hr light and dark cycles. Neuronal tissues (spinal cord, sciatic nerve, brain, and optic nerves) were collected from cervical dislocated mice.

### Constructs of MBP and His tagged Myo Va proteins

Myo Va head, neck and tail regions tagged with maltose binding protein (MBP) were described previously [11]. A Myo Va head construct encoding amino acids 41–505 was generated with Xho I -Eco RI [6]. This construct was digested with Xho I- Bst BI to generate 41–326 and Bst BI-Eco RI to generate 327–505 constructs [6]. To generate a neck region construct (amino acids 654–1402), Sal I was used. A construct encoding amino acids 640–881 was generated with Bmg BI- Psp XI from a Myo Va cDNA [11]; the 718–799 construct was PCR amplified with primer sets of 5′-GCA TGA ATT CCA GAA AGA TGT CCT TAG T-3′ and 5′-GCA AGC TTC TGA ATG GTG ATG GCA GC-3′; a 800–881 construct was PCR amplified with primer sets of 5′-GCA TGA ATT CAG ATA TGT CAG AGG GCA C-3′ and 5′-GCA AGC TTT CGA GCC AGC CAG CCT CTC-3′ and chicken Myo Va as a template [11]. All of the fragments were gel purified and cloned in-frame with an MBP sequence into a pMAL vector (New England Biolabs, MA) to tag the proteins with MBP. Myo Va tail regions spanning amino acids 952–1852 were digested with Eco RI, 952–1256 and 1257–1852 were generated by digesting 952–1852 with Sac I and Eco RI, and cloned into pQE vectors (Qiagen, Valencia, CA) to tag the proteins with hexa-His peptide (See Fig. S1F-E for protein expression).

### Construction of Myc-tagged NF-L domain deletions

A full-length NF-L-myc tagged construct [16] was digested with Nde I- Acc 65 I (1-369 amino acids), and Nde I- Bgl II (1–243) was end filled and ligated in frame with the C-terminal Myc-tagged pET11a expression construct (Novagen, Gibbstown, NJ). Full-length NF-L construct was also digested with Nde I-Pvu II (1–93 amino acids), and Pvu II-Bgl II (94–243), end-filled and ligated in frame with the N-terminal Myc-tagged pET11a expression vector (for protein expression, see Fig. S1G).

### Preparation of bacterial lysates and purification of Myo Va constructs

MBP tagged-Myo Va constructs and NF-L-Myc tagged constructs were transformed into the BL21-DE bacterial strain. Cultures were grown to 0.6 OD and induced with IPTG to overexpress proteins for 1 hr at 37°C. Cultures were centrifuged and the pellets were suspended in a 50 mM sodium phosphate buffer pH 7.0 containing 1% SDS and 1 mM EDTA. Cells were subjected to freeze-thaw in a dry ice-ethanol bath (3 times), sonicated and centrifuged. Clear supernatants were assayed for protein, immunoblotted with either MBP or NF-L (NR-4 or 21.4) or Myc (9E10) antibodies. Some of the MBP-Myo Va constructs were affinity purified on amylose columns (New England Bio Labs, MA). Bacterial cells after induction were lysed in a column buffer containing 20 mM Tris-HCl, pH 7.4, 200 mM NaCl and 1 mM EDTA by freeze thawing in a dry ice ethanol bath (3X). The lysates were centrifuged and the pellets were dissolved in the column buffer (20 mM Tris-HCl, pH 7.4, 200 mM NaCl and 1 mM EDTA) with 6M-guanidine hydrochloride, dialyzed in TBS buffer (20 mM Tris/HCl, pH 7.4 containing 150 mM NaCl) over night at 4°C, and centrifuged. Clear supernatants were then loaded on a 5 ml amylose column (30 ml/hr), washed and the proteins were eluted with 10 mM maltose in column buffer.

### Purification of NF-L from mouse spinal cords

NF-L was purified from mouse spinal cord NF pellet as described earlier [22] with modifications. Briefly, mouse spinal cords were homogenized in a buffer (1∶5 W/V) containing 20 mM Tris-HCl, pH 6.8 with 1 mM each of EDTA, EGTA, DTT and 100 mM NaCl, 0.2 mM PMSF, leupeptin (5 µg/ml) and 1% Triton X-100, and centrifuged at 20,000 g for 45 min. The pellet (P1) that has NFs was homogenized in the same buffer with 1 M sucrose, and centrifuged as indicated above for 30 min to obtain NF enriched pellet (P2). This step was repeated 3 more times to obtain NF pellet (P5) that is highly enriched in NFs. The NF pellets were dissolved in 8 M urea and subjected to 7.5% SDS-PAGE on 17 cm gels with molecular weight markers. The gels were stained with 0.3 M Zinc Chloride as described previously [23], the transparent protein band of 70-kDa NF-L was cutout, washed in 0.25 M EDTA, electroeluted in 1X SDS-gel running buffer in a dialysis bag at 20 mAmp overnight at 4°C. The electroelution was carried out for another hr without SDS in the same buffer, eluted protein was dialyzed overnight in a 50 mM Ammonium bicarbonate, pH 8.0 buffer. Eluted NF-L concentration was determined by BCA protein assay, analyzed on SDS-PAGE to observe a single band of 70-kDa that is stained with Coomassie Blue, immunoblotted with NR-4 and 21.4 antibodies to confirm that the protein is NF-L (see Fig. S1A, lanes 1, 3, 5 and 7).

### Preparation of Triton X-100 insoluble cytoskeletal fractions enriched in neurofilaments

Brain, optic nerve, spinal cord, and sciatic nerves from wild type (WT) and NF-L null mice were dissected and cytoskeletal preparations enriched for NFs were made according to [16]. Briefly, tissues were homogenized in a cytoskeletal extraction buffer (50 mM Tris-HCl, pH 6.8; 200 mM NaCl, 1% Triton-X-100, 20% glycerol, and 1 mM EDTA), centrifuged, and the pellets containing the cytoskeletal fractions were suspended in the same buffer, sonicated, and analyzed for protein content.

### Isolation of organelle-containing cytoskeletal scaffolds from optic nerve

Optic nerves were homogenized in a buffer containing 50 mM Tris-HCl, pH 7.4, 0.25 M sucrose, 1 mM each of EDTA, EGTA and DTT [24], centrifuged at 7000 g for 20 min at 4°C, and supernatants were saved. Pellets containing organelles associated with cytoskeleton were homogenized again in the same buffer and sonicated. Equal amounts of cytoskeletal and supernatant proteins were immunoblotted with antibodies to cytoskeletal proteins and organelle markers listed below.

### Immunoblot analysis of bacterial and mouse extracts

Bacterially expressed Myo Va mutant proteins, and NF-L mutant proteins, purified or cytoskeletal preparations containing NF-L, and total tissue extracts from mouse neuronal tissues (WT, NF-L null and DL20J mice) were prepared according to [16]. Proteins were fractionated on polyacrylamide gels containing SDS, transferred to nitrocellulose membranes, blocked in 3% non-fat dry milk, and incubated with either anti-MBP-HRP (New England Bio Labs, MA), or other antibodies including (source in parentheses) NF-L (NR-4), hexa-His, α, β, βIII tubulin, synaptophysin, and actin (Sigma Chemical Co, St. Louis, MO); 21.4 [25]; Myc (9E10, Santa Cruz Biotech, CA); Myo Va [16]; calnexin (Stressgen Bioreagents, Canada); PSD-95 (Millipore, CA); Rab-5 (Abcam, MA), and Rho B (Cell Signaling, MA). After washing, the membranes were incubated with the corresponding HRP-conjugated secondary antibodies and immunoreactive bands were detected with ECL reagent (GE Healthcare, NJ). Images were scanned, and bands were quantified with a multigauge program (Fuji film, Japan).

### Co-immunoprecipitation of NF-L and Myo Va mutant proteins

Using purified MBP as a standard (NEB, MA), we quantified the amount of MBP-tagged Myo Va mutant proteins for each expressed construct on immunoblots with anti-MBP antibody. Equal amounts of protein corresponding to each MBP-Myo Va construct (0.5–2 µg) and purified or Myc-tagged NF-L (0.5–2 µg) were mixed for 1 hr in 1X RIPA buffer for co-IP assays. Antibody directed against NF-L (NR-4 or 21.4 or 9E10 for Myc tag) was then added and the mixture was incubated for 1 hr at room temperature (RT). Protein A/G Sepharose beads were added and incubated for another 1 hr at RT. Immunocomplexes were centrifuged and the precipitates were washed (3X) with high salt solution (50 mM Tris-HCl, pH 7.4; 500 mM NaCl; 1 mM EDTA; 0.1% NP40; 0.02% SDS; 0.02% NaN3), dissolved in Laemmli buffer, fractionated on polyacrylamide gels along with 5–10% of the supernatant from the IPs and the input. Gels were transferred to nitrocellulose membranes and immunoblotted with MBP and NF-L antibodies as indicated above. To take equimolar concentrations of the protein, bacterial extracts containing NF-L and MBP-tagged Myo Va proteins were fractionated on gels along with known concentrations of BSA. The Coomassie stained gels were scanned and protein bands of NF-L, MBP-Myo Va and BSA were quantified using the multigauge program. To compare two proteins based on the number of amino acids each protein has, we calculated the molecular weight (MW) of the proteins and the MW of the bigger protein was divided by that of the smaller protein. The resultant value was multiplied by the amount of protein taken for the smaller construct to obtain the equimolar concentration of the larger protein. To achieve an excess amount of NF-L for IPs, we took a 2–3 fold greater amount of NF-L relative to the MBP-Myo Va constructs.

### Actin activated Myo-Va-ATPase assay

Myo Va enriched from vesicles was prepared from mouse brain as described previously [26]. Briefly, mouse brains were homogenized (1∶4 W/V) at 4°C in 40 mM HEPES, pH 7.8, 10 mM K-EDTA, 5 mM ATP, 2 mM DTT and 2 mM PMSF in a Glass-Col homogenizer (20 strokes at 70 setting). Homogenates were centrifuged at 40,000 g at 4°C for 40 minutes. The salt concentration of the supernatant (S-1) was raised to 0.6 M NaCl, and the solution was incubated on ice for an hour followed by centrifugation at 150,000 g for 2 hrs to isolate Myo Va enriched pellet. The pellet (P-2) was suspended in a buffer (50 mM Tris/HCl pH 7.5) and Myo Va-ATPase assay was carried out with Novus Biological kit (Littleton, CO) according to manufactures suggestions and an excess amount of purified actin (5 µg from bovine skeletal muscle) or NF-L (10 µg, from mouse spinal cord) was added in a separate assay.

### Blot overlay assays

Blot overlay assays were conducted as described previously [16]. Briefly, purified vimentin (Cytoskeleton, Denver, CO), desmin (Prospec, Rehovot, Israel), actin (Sigma, St. Louis, MO) or recombinant or cytoskeletal preparations from brain, optic nerve, spinal cord, and sciatic nerves were fractionated on 7% acrylamide gels containing SDS. Proteins on these gels were transferred to nitrocellulose membranes, blocked, incubated with MBP-tagged Myo Va constructs overnight at 4°C, washed, incubated with α-MBP-HRP antibody (New England Biolabs, MA) and washed. Immunoreactive signals were detected with ECL reagent.

### Morphometric analysis and density measurements for ER

Mice were perfused and optic nerves were fixed and embedded for electron microscopy as described previously [16]. Sections were taken at the 2-mm level of optic nerve, imaged, and photographed at 25K magnification. ER profiles were counted from axons of caliber sizes representative of the whole nerve using Bioquant software (Nashville, TN). The total numbers of ER were divided by the axon area to obtain the density of organelles in a given axon. To analyze the peripheral distribution of ER vesicles, cross sections of WT, NF-L null, DL20J and dilute lethal control (DLC) optic axons were imaged at 25K and printed at 50K magnification. ER vesicles that were within 100 nm from the periphery of the axolemma were considered peripheral and those more centrally located in the axon were considered non-peripheral. Optic axons that were larger than 1 µm were taken for analysis from WT (n = 81), NF-L null (n = 63), DLC (n = 69) and DL20J (n = 100) mice.

## Results

### Myo Va binds to NF-L through its motor head domain

**Figure 1:**
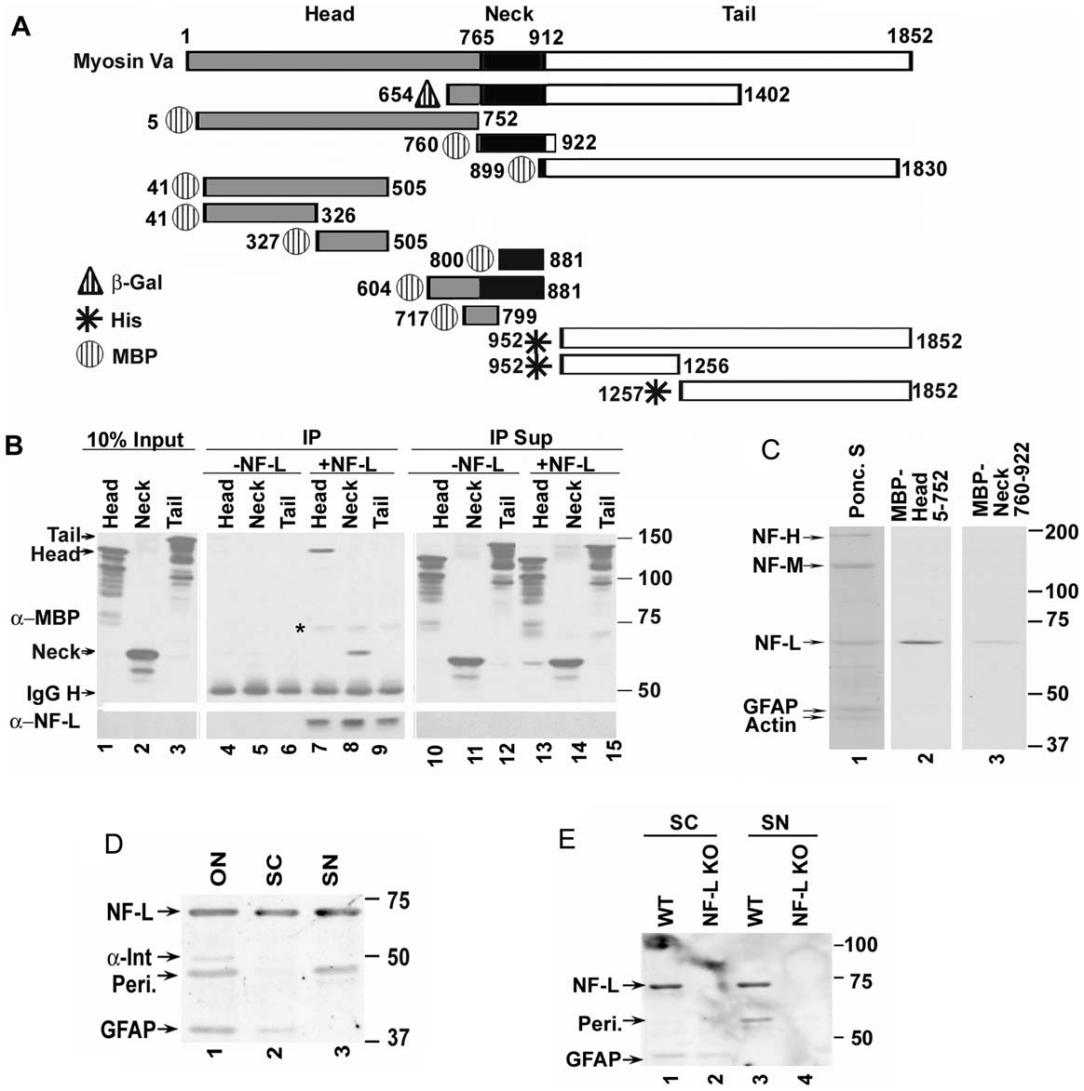
Schematic representation of Myo Va deletion constructs. (A). Amino acid numbers correspond to the mouse dilute gene product [6]. The three domains of Myo Va are color coded (head-grey; rod-black and tail-white). The mutant proteins are either tagged with MBP (Hatched-circle) or hexa-His-peptide (star) or 3-kDa β-gal peptide (hatched-triangle). Constructs 5–752, 760–922 and 899–1830 are from chicken [11]. The construct tagged with β-gal-654-1402 is of human origin [15], and the tail clones originate from the mouse sequence [6]. Clones 718-799 and 800–881 are PCR products from chicken Myo Va [11]. (B) *Relative binding of Myo Va tail, head and neck regions to NF-L.* Myo Va head, neck and tail domains were incubated with NF-L, co-immunoprecipitated with NF-L antibody (NR-4), immunoprecipitates (IPs) and supernatants (Sup, 10%) were immunoblotted with anti-MBP and NF-L antibody (NR-4) and immunoreactive bands were detected with ECL reagent. The molecular weight (MW) of Myo Va head domain-120, neck-60 and tail is 140-kDa. *-indicates non-specific binding. (C) Myo Va head region shows more binding to NF-L compared to the neck region in blot overlay assays. Cytoskeletal fractions from spinal cord fractionated on 7% acrylamide gels containing SDS, transferred to nitrocellulose membranes, blocked with 3% milk, incubated with head and neck constructs tagged with MBP at equimolar concentrations, immunoblotted with anti-MBP-HRP antibody and immunoreactive bands were detected with ECL reagent. Ponc. S: Pnoceau S. (D) Myo Va head region binds to NF-L, α-internexin, peripherin and GFAP in blot overlay assays. Cytoskeletal fractions from optic nerve (ON), spinal cord (SC), and sciatic nerve (SN) were fractionated, and the Myo Va head domain binding activity was detected by blot overlay assay as described in panel C. (E) Myo Va head region binding to NF-L is specific since NF-L deficient cytoskeletal preparations do not show NF-L binding activity. Cytoskeletal fractions from WT and NF-L null (NF-L KO) mice spinal cord (SC) and sciatic nerve (SN) were fractionated and blot overlay assays were performed to detect Myo Va head domain binding activity as described in panel E. The positions of the bands on the membranes are indicated with arrows in all panels. Anti-MBP: α-MBP; anti-NF-L:α-NF-L.

To identify which of the Myo Va mutants bind to full length NF-L, we co-immunoprecipitated (co-IP) each of these Myo Va mutant proteins (Fig. 1A and Fig. S1A-E) in equimolar quantities with an excess of NF-L, using the antibodies NR-4 or 21.4 for purified NF-L and 9E10 for Myc tagged NF-L (Fig. S1F). Analysis of the immunoprecipitates (IPs) by Western blotting using MBP antibody indicated that both the Myo Va head domain (5-752, Fig. 1B, lane 7) and the neck region (760-922, Fig. 1B, lane 8) bound to the NF-L subunit (Fig. 1B), while the tail region (Fig. 1B, lane 9) failed to show binding activity under these conditions. Our IP assays also demonstrated that the head region of Myo Va bound to NF-L more than the neck domain (compare lane 7 with 8 in Fig. 1B). The latter construct was part of the very long Myo Va construct (654-1402; see Fig. 1A) previously used to show binding to NF-L [16].

The Myo Va cargo binding region is present in the tail region where it binds to melanosomes [27]. The tail region of the Myo Va (952-1852) construct, however, did not bind to NF-L in our co-IP experiments (Fig. 1B lane 9). To exclude the possibility that tagging the tail region with MBP interferes with its binding, we tagged the tail constructs with hexa-His peptide at the N-terminus of the various Myo Va mutants, and also divided the tail region into 2 parts (952-1256 and 1257-1852 amino acids) (Fig. 1A & Fig. S1E). Co-IP studies indicated that these tail mutants have no binding activity with full-length NF- L whereas the Myo Va head bound to NF-L in the same experiments (data not shown).

We confirmed the specificity of Myo Va head binding to NF-L, by co-immunoprecipitating NF-L with either MBP-Myo Va-Head (5-752), purified MBP alone, or bovine serum albumin (BSA). MBP-Head did co-immunoprecipitate with NF-L, while MBP alone or BSA (data not shown) showed no binding activity, indicating that the MBP-Myo Va head interaction with NF-L is specific.

We next used spinal cord cytoskeletal fractions in blot overlay assays [16] to confirm our IP studies and to investigate whether the head and neck regions of Myo Va have similar binding affinities towards NF-L. After SDS-PAGE, cytoskeletal proteins were transferred to nitrocellulose membranes and subjected to blot overlay assays with Myo Va head and neck regions at equimolar ratios. The blot overlay results demonstrated that the Myo Va head region showed more binding activity (Fig. 1C, lane 2) with NF-L compared to the neck region (Fig. 1C, lane 3), which had been previously shown to bind a part of a larger construct (954-1402) [16].

In similar blot overlay analyses, we confirmed that NF-L is a major ligand for the Myo Va head domain (5-752) in optic and sciatic nerves and spinal cords of WT mice (Fig. 1D). Upon longer exposure of the autoradiographs, however, we found that three additional IF proteins, α-internexin, peripherin, and GFAP, also bound to the Myo Va head in optic nerve (Fig. 1D, lane 1). Moreover, Myo Va bound GFAP in spinal cord (Fig. 1D, lane 2), and peripherin in sciatic nerves (Fig. 1D, lane 3). These results suggest that Myo Va head binds to different neuronal IFs.

To further demonstrate the specificity of Myo Va head binding to NF-L, we subjected cytoskeletal fractions of spinal cord (Fig. 1E, lanes 1, 2) and sciatic nerves (Fig. 1E, lanes 3,4) from WT (Fig. 1E, lanes 1, 3) and NF-L null (Fig. 1E, lanes 2, 4) mice to blot overlay assays with the Myo Va head region. The results revealed that spinal cord and sciatic nerve cytoskeletal fractions from WT mice showed strong NF-L binding activity (Fig. 1E, lanes 1, 3), while the same activity was absent in NF-L null mice (Fig. 1E, lanes 2, 4). Moreover, both WT and mutant mouse spinal cords showed GFAP binding activity with the Myo Va head region (Fig. 1E, lanes 1, 2). These results suggest that the interactions between Myo Va and NF-L are specific. It was shown previously that loss of NF-L in sciatic nerves dramatically reduces levels of axonal peripherin [28]. As expected, therefore, we were unable to detect binding of Myo Va to peripherin in sciatic nerves of NF-L null mice (Fig. 1E lane 4); however, the binding of Myo Va to peripherin was readily detected in sciatic nerve fractions of WT mice (Fig. 1E, lane 3).

**Figure 2:**
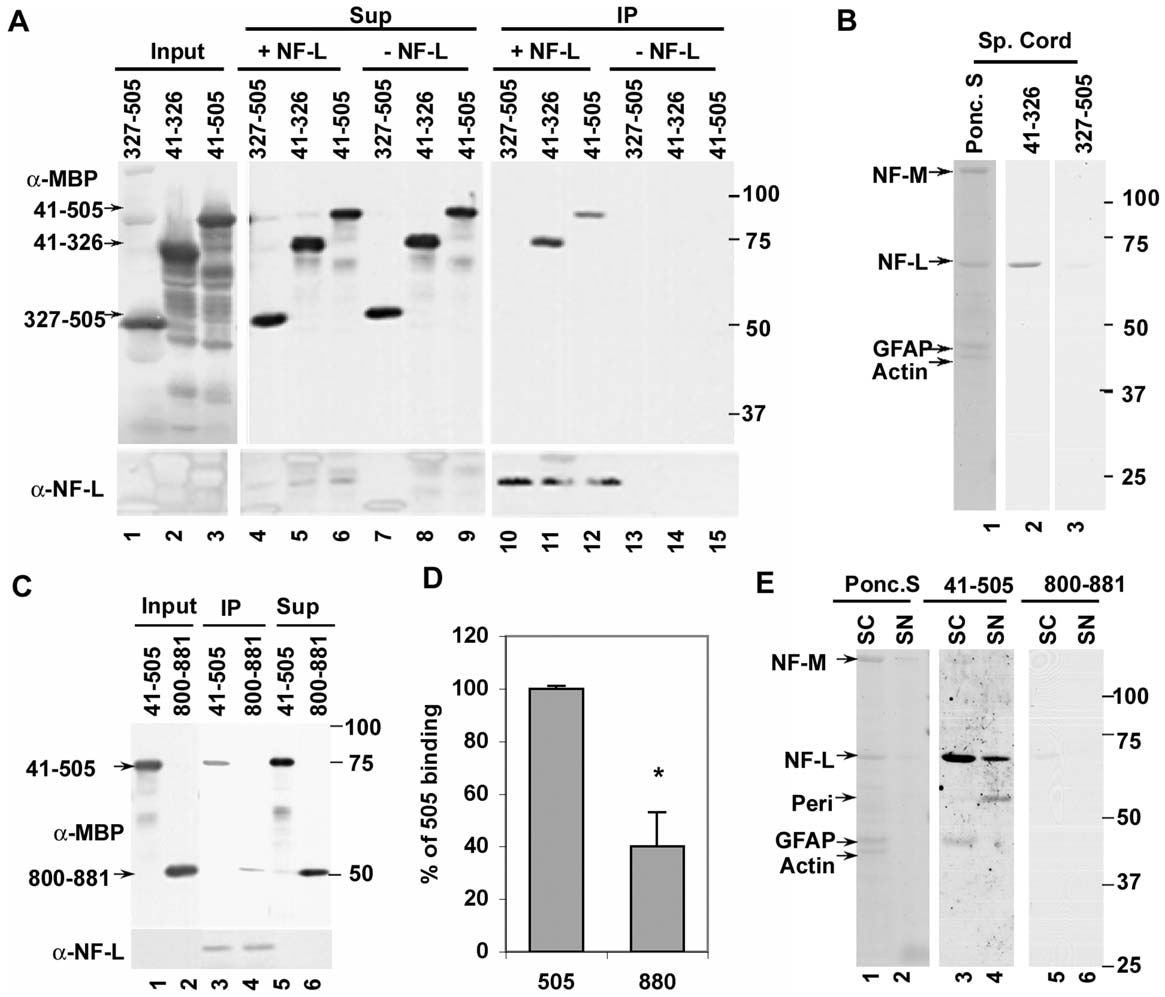
Myo Va motor domain binds to NF-L. (A) The N-terminal motor domain (41–326) of Myo Va binds to NF-L in co-IPs. Myo Va head domain (41–505), N-terminal head domain (41–326), C-terminal head domain (327–505) proteins were incubated with or without NF-L and immunoprecipitated with NF-L antibody (NR-4), IPs and Sups (10%) were immunoblotted with anti-MBP-HRP, and anti-NF-L antibody (NR-4). The immunoreactive bands were detected as described in Fig. 1B. Myo Va head domain (41–505) is 95-kDa, 41-326 is 75-kDa and 327–505 is 52-kDa. (B) The N-terminal domain of Myo Va (41–326) binds to NF-L in blot overlay assays at equimolar concentrations with spinal cord cytoskeletal fractions. Lane 1 Ponceau S stain (Ponc. S.) of SC cytoskeletal fraction, SC cytoskeletal fractions in lane 2 and 3 were incubated with 41–326 and 327–505 of Myo Va head mutants and blot overlay assays were performed as described in Fig. 1C. (C&D) Myo Va head domain (41–505) shows significantly more binding to NF-L in co-IPs (C&D, *-p<0.011) and blot overlay assays (E) compared to neck region (800–881). The MW of 800–881 is 50-kDa. Error bars represent SEM. Myo Va head domain (41–505) and neck region (800–881) were separately incubated with or without NF-L and immunoprecipitated with NF-L antibody (NR-4). IPs and Sups (10%) were immunoblotted with MBP-HRP and NF-L antibodies in panel D. (E) Cytoskeletal fractions from spinal cord (SC, Fig. 2E, lanes 1, 3&5) and sciatic nerves (SN, Fig. 2E, lanes 2, 4&6) of WT mice were transferred to nitrocellulose membranes and Ponceau S stained (Fig. 2E, lanes 1&2), and blot overlay assays were performed with Myo Va head (41–505, lanes 3&4) and neck regions (800–881, lanes 5&6) at equimolar concentrations. The positions of proteins in all membranes were indicated with an arrow. Anti-MBP: α-MBP; anti-NF-L:α-NF-L.

In the above studies, the Myo Va head domain construct used spans amino acids 5-752. We constructed 3 additional mutants of the head region (41-505, 41-326 and 327-505), which were tagged with MBP (Fig. 1A) to further define the NF-L binding site on this region. Co-IPs at equimolar ratios with these Myo Va constructs demonstrated that the 41-505 (Fig. 2A, lane 12) and 41-326 (Fig. 2A, lane 11) but not the 327-505 (Fig. 2A, lane 10) region of Myo Va bound to NF-L. These results confirm that Myo Va binds NF-L principally through the N-terminal region of the head domain, which contains the ATP binding region and the motor domain. To confirm the differential binding activities of these two constructs seen in IP assays with NF-L, we subjected cytoskeletal fractions from spinal cord to blot overlay assays with 41-326 and 327-505 in equimolar amounts. Only 41-326 bound to NF-L (Fig. 2B, lane 2), whereas 327-505 failed to show binding activity (Fig. 2B, lane 3) in spinal cord fractions.

Because our studies previously demonstrated binding activity of a Myo Va construct containing the neck region (654-1402) [16], we constructed three additional neck mutants (Fig. 1A). These neck mutants were highly unstable, which made binding assays with these constructs very difficult to perform. Additional co-IPs to investigate the relative binding activity of head and neck constructs revealed that the Myo Va head region (41-505) bound to NF-L under conditions where 800-881 showed much less binding activity (Fig. 2C, compare lanes 3&4). The quantitative data from multiple experiments demonstrate that the Myo Va motor domain (41-505) showed significantly more binding activity compared to the 800-881 construct (Fig. 2D, p<0.015). Additional blot overlay assays also demonstrated that, at equimolar concentrations, the 41-505 construct showed more binding activity to NF-L (Fig. 2E, lanes 3,4) compared to 800-881 fragment (Fig. 2E, lanes 5&6) in spinal cord (Fig. 2E, lanes 1, 3 and 5) and sciatic nerve (Fig. 2E, lanes 2, 4 and 6) fractions.

### Myo Va motor domain binds to the amino terminal end of the NF-L rod domain

**Figure 3:**
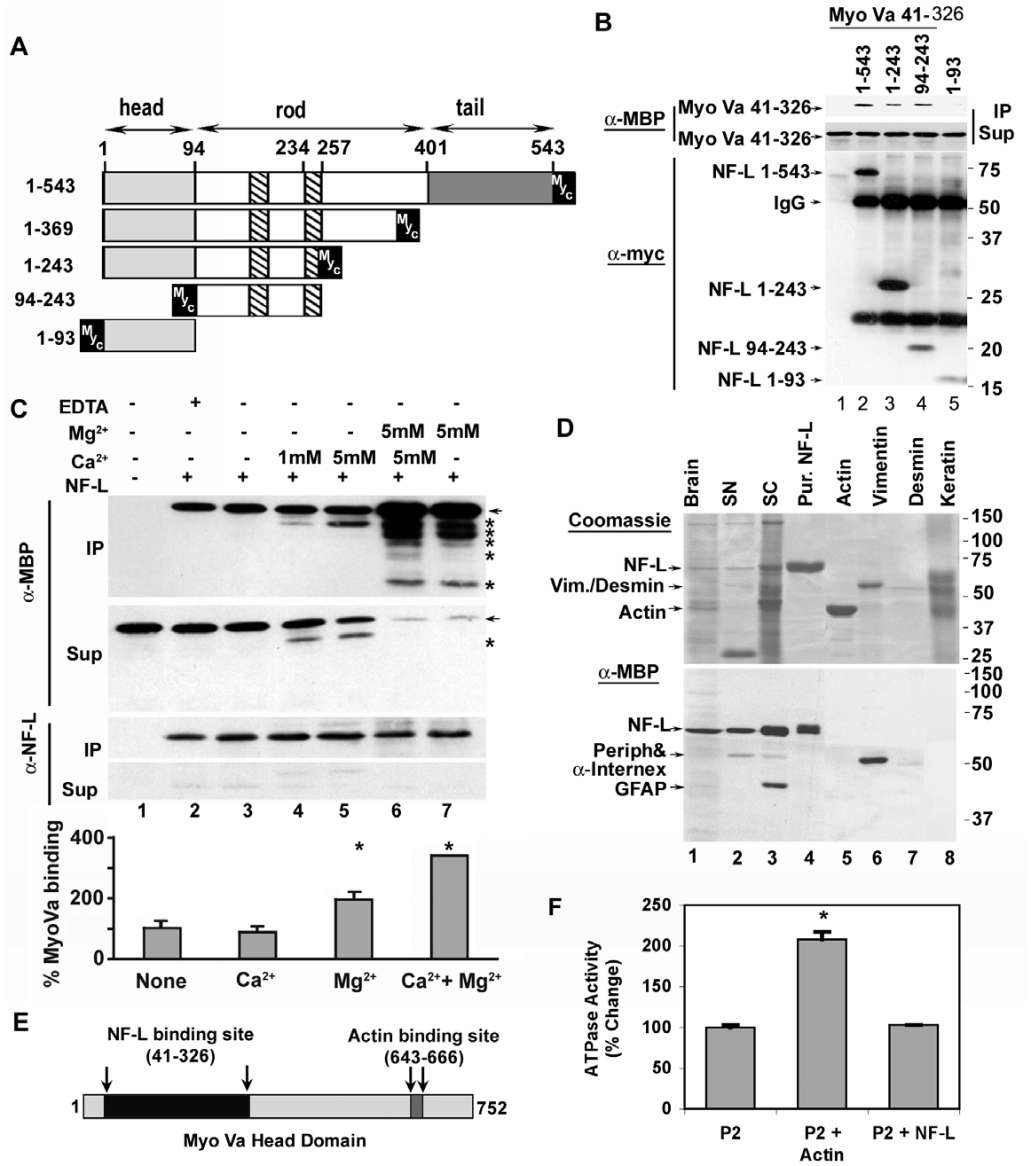
The N-terminal rod domain of NF-L binds to the motor domain of Myo Va. (A) Schematic representation of NF-L subunit domains and the deletions. Amino acids are numbered according to [31]. See materials and methods for construction of NF-L domain deletions. B. The amino-terminal motor domain of Myo Va (41–326) binds to the N-terminal rod domain of NF-L. Myo Va constructs were MBP-tagged while NF-L was myc-tagged. Co-immunoprecipitation of NF-L deletion constructs with Myo Va motor domain (41–326). Myo Va was incubated without NF-L (lane 1), with full length NF-L 1-543 (lane 2); NF-L 1-243 (lane 3); NF-L 94-243 (lane 4) and NF-L 1-93 (lane 5) were immunoprecipitated with Myc antibody (9E10), IPs and Sups (10%) were immunoblotted with anti-MBP or anti-Myc antibodies. The molecular weights of NF-L 1-543, 1-243, 94-243 and 1-93 are 68, 27, 20 and 17-kDa respectively. (C) Magnesium ions potentiate Myo Va and NF-L binding. Myo Va motor domain (41–326) was incubated with NF-L (Full length) and immunoprecipitated with α-Myc antibody. The resulting IPs and 5% of sups were immunoblotted with α-MBP-HRP or α-Myc Abs. Addition of magnesium (5 mM) increased binding (see the graph in 3C) while addition of magnesium and calcium (5 mM) further increased the binding of NF-L to Myo Va (compare lane 6 with 5 and 7). Arrows indicate the position of Myo Va motor domain (41–326) of 75-kDa protein. The *-indicates proteolytic fragments of 41–326 construct generated with incubation of calcium and magnesium in both IP and supernatant fractions in lanes 4&5 while these are seen only in IP fractions of lanes 6&7. (D). Myo Va head domain binds to different IF family members. Cytoskeletal preparations from brain (lane 1), sciatic nerve (lane 2), spinal cord (lane 3), purified NF-L (lane 4), purified actin (lane 5), purified vimentin (lane 6), purified desmin (lane 7) and fractionated keratin from human skin (lane 8) were separated on gels and transferred to membranes. Blot overlay assays were performed on membranes with MBP-Myo Va (41–326) protein and immunoblotted with α-MBP-HRP antibody. Anti-MBP: α-MBP; anti-NF-L: α-NF-L and anti-myc: α-myc. *(E) Schematic representation of NF-L and Actin binding sites on Myo Va head domain* (1-752). The amino acids are numbered according to [6]. (F). *Actin activates Myo Va-ATPase activity while NF-L does not.* Myo Va was fractionated from adult mouse brain according to [26] (P2 fraction enriched in vesicular Myo Va) and incubated with either actin or NF-L and assayed for ATPase activity. Addition of actin to P2 fraction activated Myo Va-ATPase activity while addition of NF-L did not. *p<0.02. Error bars represent SEM in all experiments.

To identify the site on NF-L to which the Myo Va motor domain binds, we constructed four Myc-tagged NF-L deletion mutants (1-369; 1-243; 1-93 and 94-243; Fig. 3A) by restriction enzyme digestion (see Methods). The physical properties of NF-L deletion constructs are described in Table 1. The molecular weights of these constructs measured on SDS-PAGE differ from the calculated molecular weights due to abnormal behavior of NF proteins on these gels [29]. The WT NF-L displayed pI values of 4-5 in the acidic range while the head domain has a basic pI of 9.8. The full length NF-L and the mutants were detected with anti-Myc antibodies (Fig. S1G).

**Table 1:** Some Physiochemical Properties of Engineered NF-L Constructs.

NF-L	Source	Amino acids	Observed Molecular Weight	Calculated Molecular Weight	Protein Characteristics		
					*Estimated* *Pl*	*Acidic* *AA %*	*Basic* *AA %*
Full length	Mouse	1–543	68-kDa	68000	4.41	22.7	14.4
Headless	Rat	65–543	54-kDa	61500	4.31	25.2	14.5
Tailless	Mouse	1–369	37-kDa	47000	5	17.9	15.7
Head + Rod	Mouse	1–243	27-kDa	28000	5	17.3	15.2
Rod	Mouse	94–243	20-kDa	20000	4.5	24	16.7
Head	Mouse	1–93	17-kDa	16000	9.8	6.46	12.9

To test which of the NF-L deletion construct would bind to the Myo Va motor domain (41-326), we performed co-IP assays. Our data indicate that Myo Va bound to all the NF-L constructs containing amino acids 94-243 in the rod domain (Fig. 3B, lanes 2–4). By contrast, construct 1-93 containing the head domain of NF-L, showed negligible binding to the Myo Va motor domain (Fig. 3B, lane 5). Therefore, our data, identify the Myo Va binding site on NF-L as the N-terminal portion of the rod domain encompassing amino acids 94–243. The binding of Myo Va to cellular cargoes is regulated by calcium and magnesium in the cell [30]. We tested whether these ions would modulate the Myo Va motor domain (41–326) binding to NF-L. Our co-IP studies in the presence of these ions indicate that Mg^2+^ enhanced binding (Fig. 3C, and the graph in C) while Ca^2+^ had an insignificant effect on Myo Va-NF-L binding (Fig. 3C, lanes 4, 5).

### Myo Va motor domain binds to multiple intermediate filaments

All IF proteins have a common structural core of 300–330 amino acid residues within the rod domain, which are flanked by variable amino- and carboxy-terminal regions [31]. To investigate possible binding of other IF family members to Myo Va motor domain, we performed blot overlay assays with the IF preparations made from mouse brain, sciatic nerve, spinal cord, purified NF-L, actin (bovine skeletal muscle), vimentin (hamster), desmin (human), and keratin from human skin. Our data indicate that the Myo Va motor domain not only binds to NF-L from brain, sciatic nerve, spinal cord and purified NF-L from mouse spinal cord as a major ligand (Fig. 3D, lanes 1–4) but also binds to GFAP (Fig. 3D, lanes 1,3), α-internexin (Fig. 3D, lane 3), peripherin (Fig. 3D, lane 2), vimentin (Fig. 3D, lane 6) and desmin (Fig. 3D, lane 7), but not to actin or keratin (Fig. 3D, lanes 5,8). As expected, actin (Fig. 3D, lane 5) did not bind to this Myo Va construct (41–326) because the construct lacks the binding site for actin (amino acids 643–666) [32].

### F-actin activates Myo Va-ATPase activity while NF-L does not

The actin and NF-L binding sites are about 300 amino acids apart on the Myo Va head domain (Fig. 3E). Because of this proximity, it is possible that NF-L and actin may compete with each other to bind to Myo Va; however, the Myo Va head construct, containing the actin binding site, did not bind to actin despite attempts using various conditions. Actin has previously been observed not to bind to the bacterially expressed full length Myo Va head protein because it does not fold properly, which is necessary for its actin binding activity (Mark Mooseker, personal communication). Our published data indicate that under *in vivo* conditions, Myo Va, NF-L and actin holoproteins form complexes and these were identified by co-immunoprecipitation assays in mouse optic axons [16].

To examine whether NF-L (purified from mouse spinal cord) would activate Myo Va-ATPase activity, we incubated fractionated Myo Va (P2 fraction, [26]) with NF-L in an ATPase assay buffer. We did not find an increase in Myo Va-ATPase activity with addition of NF-L, while addition of F-actin led to an increase in Myo Va-ATPase activity in a parallel assay. These data indicate that NF-L binding to Myo Va, by itself, does not activate Myo Va-ATPase activity in our assay conditions (Fig. 3F).

### Loss of NF-L and NF-networks in axon leads to reduced scaffold function and lowered axonal organelle content

**Figure 4:**
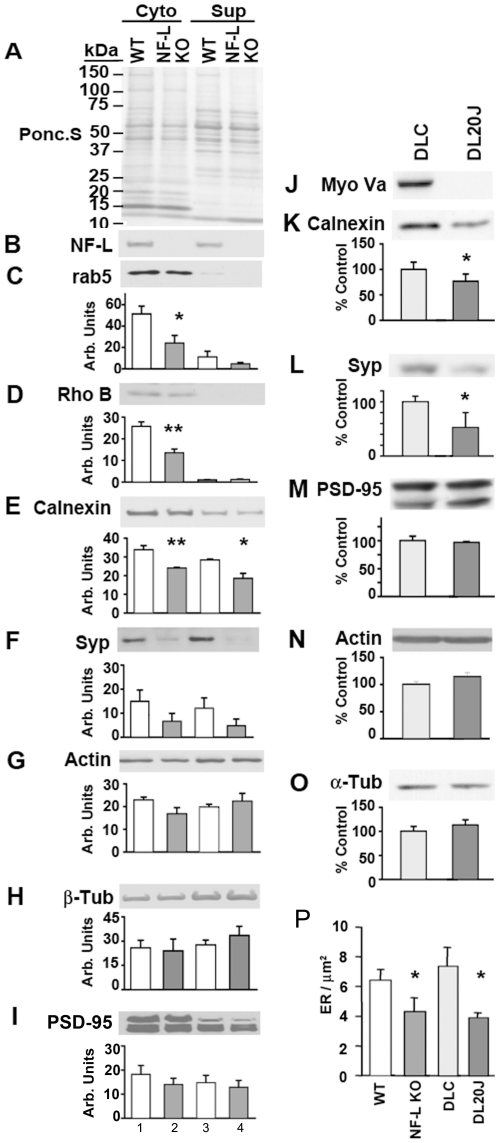
NF-network-dependent association of cellular organelles is reduced in NF-L null optic axons. Cytoskeletal (lanes 1&2), and supernatant fractions (lanes 3&4) from WT (lanes 1&3) and NF-L null (lanes 2&4) optic nerves were resolved by SDS-PAGE, transferred to membranes, Ponceau S stained (A), immunoblotted with NF-L (B), Rab-5 (C), Rho B (D), calnexin (E), Synaptophysin (SYP, F), actin (G), tubulin (H), and PSD-95 (I) antibodies. Densitometric quantification of signals from immunoblots in panels C-F indicate significantly reduced amounts of Rab-5 (C), Rho B (D), calnexin (E), Synaptophysin (SYP, F), in NF-L null cytoskeletal fractions compared to controls while actin (G), tubulin (H), and PSD-95 (I) were not decreased in the same fractions. (n = 6 for each genotype of 5–6 month old mice). (J). Total extracts of optic nerves from DLC (dilute lethal control), and DL20J mice were immunoblotted with Myo Va, calnexin, Synaptophysin (SYP), actin, tubulin and PSD-95 antibodies. Quantitation data for calnexin (K), synaptophysin (SYP; L), PSD-95, actin and tubulin is shown in M-O. (P). Morphometric analysis indicate reduced density of ER vesicles in NF-L null and DL20J optic axons (n = 4 for 5–6 month old WT and NF-L null, and 17 day for DL20J and DLC). *p<0.05, and **p<0.01. Error bars represent SEM in all experiments.

IFs serve as cytoskeletal scaffolds in the nucleus and cytoplasm of higher metazoans [33], [34]. Myo Va binds to synaptic vesicles, ER, Golgi, endosomes and mitochondria [16], [35], and also to the NF-L subunit. Loss of NF-L leads to reduced Myo Va in axons [16]. We hypothesized that if the cellular organelles are anchored within axonal NF networks through Myo Va to stabilize their positions in the axon, then loss of Myo Va-NF-L interactions would be expected to reduce organelle associations with NF scaffolds and lead to reduced organelle content as organelles resume axonal transport ultimately back to the perikaryon for degradation. To test this hypothesis, we homogenized the optic nerves of NF-L null mice in an isotonic 0.25 M sucrose buffer, and centrifuged at low speed (7000 g) to isolate the organelles that associate with NF-networks under isotonic conditions. As predicted, a major proportion of organelles analysed (ER, synaptic vesicles, endosomes) fractionated into the cytoskeletal fraction, including the majority of calnexin (a marker of ER), Rab-5 and Rho B (endosomal markers), and synaptophysin (SYP, a synaptic vesicle marker) (Fig. 4A-F), implying either direct associations between NFs and vesicular structures (ER, synaptic vesicles, endosomes) or entrapment of these organelles within the NF network. By contrast, in NF-L null mice, the content of Rab-5, Rho B, calnexin, and SYP were significantly lowered compared to the cytoskeletal fractions from WT mice (Fig. 4A-F). However, the content of actin, tubulin and PSD-95 between WT and NF-L null axons was not significantly altered (Fig. 4G-I). The reduced content of calnexin, Rab-5, Rho B and SYP in total homogenates of NF-L null optic extracts is most consistent with a failure to anchor organelles in the axon and with their ultimate retrograde transport back to the perikarya for degradation (data not shown).

### Loss of Myo Va also reduces organelle markers in dilute lethal (DL20J) optic axons

Myo Va associates with NFs, and loss of NFs leads to a 50% reduction in axonal Myo Va (16) in NF-L null optic axons. Moreover, Myo Va is involved in the transport of synaptic vesicles, ER, and other membrane-bound organelles [35]. We tested the possibility that loss of Myo Va also would lead to reductions in organelle markers similar to those observed for NF-L null axons (Fig. 4A-F). Our immunoblot analyses of optic nerve extracts from the Myo Va mutant (DL20J) mice demonstrate that calnexin and synaptophysin (SYP) were significantly reduced compared to littermate dilute lethal control (DLC) axons (p<0.05) (Fig. 4J-L), but actin, α-tubulin, and PSD-95 were not altered (Fig. 4M-O).

### Loss of NF-L or Myo Va leads to reduced content and peripheral distribution of ER vesicles in the axon

If NF-L-Myo Va binding cage and/or anchor organelles to maintain a relatively uniform organelle distribution throughout the axoplasm, we hypothesized that loss of the NF-L or Myo Va would reduce the content of organelles and possibly alter their distribution within NF-L and Myo Va mutant axons. To test this possibility, we performed electron microscopy of fixed optic axons of NF-L null and DL20J mice, and further morphometric analysis by counting clear ER vesicles in these mutant axons and calculating the density of ER (see methods). Our morphometric analysis of NF-L null or DL20J optic axons demonstrated significantly lower numbers of smooth ER, compared to controls (Fig. 4P).

**Figure 5:**
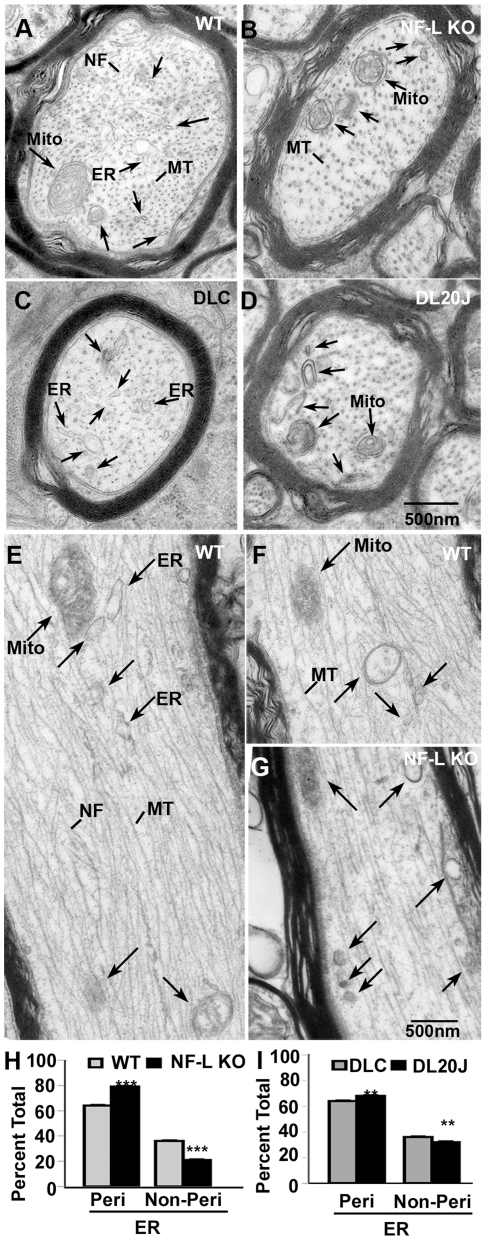
Loss of NF-L and Myo Va leads to increased peripheral distribution of ER vesicles in optic axons. In cross sections of WT (A), and dilute lethal control (DLC) (C) axons a clear tubulo-vesicular and vesicular profiles distribute diffusely in the radial dimension (A&C) but are more peripherally distributed in NF-L null (B) and DL20J (D) optic axons (see arrows in A–D). In comparison to WT axons (E, F, see arrows), cellular organelles in NF-L null optic axons (G) are peripherally distributed along channels beneath the axolemma in longitudinal sections (see the arrows in G). Quantitative measurements of distribution of organelles within 100 nm from periphery of axons that are larger than 1 µm demonstrate that increased peripheral distribution of ER in both NF-L null (H) and DL20J (I). (n = 5 animals for 6-month old WT and NF-L null, and 17 day DL20J and DLC). **p<0.01, and ***p<0.001. Error bars represent SEM. NF: neurofilaments; MT: microtubules; Mito: mitochondria; Peri: peripheral; Non-peri: non-peripheral.

Moreover, we observed that clear vesicular organelles normally distribute uniformly throughout the three-dimensional space of axons in WT mice (Fig. 5A, C, E, F). Consistent with the predominant association of ER and endosome markers with the NF cytoskeleton (Fig. 4), the organelles (ER, endosomes and mitochondria) were frequently flanked on both sides by NFs, which seemed to contact the organelles, or even enclose the vesicular structures, as described previously for melanosomes caged by vimentin filaments in non-neuronal cells [36]. By contrast, in NF-L null optic axons, these organelles were redistributed to the periphery of the axon and lined up in channels in the subaxolemmal area (Fig. 5B, G). We also observed a similar redistribution of ER, endosomes and mitochondrial profiles in DL20J optic axons (Fig. 5D) compared to DLC axons (Fig. 5C). Our morphometric quantitative measurements confirmed the redistributions identified by visual inspection (Fig. 5H, I). ER located within 100 nm from the axolemma was scored as having a peripheral distribution. Organelles more centrally located were scored as non-peripheral. Our data on cross-sectioned axons (>1 µm) from WT (n = 81), NF-L null (n = 63), DLC (n = 69), and DL20J (n = 100) mice demonstrated a significant redistribution of ER toward the periphery of axons in NF-L null (Fig. 5H) and DL20J mice (Fig. 5I) compared to their controls, while total axonal content was lowered similarly in NF-L null and DL20J mice (Fig. 4P). The effect on the peripheral distribution of ER in Myo Va mutant axons was less prominent though similar compared to that in NF-L null axons.

### Loss of NF-L does not alter synthesis or translocation rates of radiolabeled proteins that contain ER vesicles undergoing fast axonal transport in vivo

To investigate whether reduced axonal Myo Va levels in NF-L null mice [16] would alter export and fast axonal transport of membranous organelles (ER, endosomes, synaptosomes) along optic axons, we analyzed the export, transport rates, and composition of cargoes (SNAP-25, p85, and p135) that are known to translocate at fast transport rates after injecting NF-L-null mice intravitreously with [^35^S]-methionine as previously described [37]. Our data indicate that the export of SNAP-25 (synaptosome marker), p85 and p135 and the rates of fast transport of SNAP-25, p85 and p135 were not significantly altered in the absence of NF-L (data not shown), where there is a 50% reduction of axonal Myo Va [16]. The distribution patterns of radiolabeled actin and tubulin proteins were also unaltered in NF-L null axons (data not shown). For both NF-L null mice and age-matched controls, peak and front wave rates were 12 and >38.4 mm/day, respectively, for p135, 16.8 and >38.4 mm/day for SNAP-25, and 12 and >21.6 mm/day for p85 consistent with previous rate estimates [37]. These results suggest that NF-L does not contribute to the export and the transport rates of fast axonal transport cargoes in optic axons.

## Discussion

Our studies identify a novel role for the binding between Myo Va and NFs as a mechanism to anchor organelles within the axoplasm of large caliber axons. We demonstrate that NF-L, and specifically the NF-L rod domain, binds directly the N-terminal motor domain of Myo Va. It is well-established that the MyoVa cargo domain binds vesicular organelles (7–9), and our morphological and fractionation data (this study and [16]) demonstrate associations among vesicular organelles, Myo Va, and NF-L. We showed that NF-L loss reduced axonal levels of both Myo Va and organelles and increased peripheral distribution of ER toward the actin-rich subaxolemmal region. Consistent with these findings, loss of Myo Va in DL20J mice similarly reduces axonal organelle number and also increases peripheral distribution of ER.

It is known that NFs in large caliber axons are organized into an extensively cross-linked network containing microtubules, and actin filaments through multiple cross- bridging proteins [38], [39]. NFs in mature axons form a highly metabolically stable stationary network maintained by a smaller pool of newly synthesized subunits continuously transported along axons in the form of dimers, oligomers, or short filaments [40]. Video microscopy analyses of axons *in vitro* show that subtypes of vesicular organelles display different kinetic behaviors ranging from very rapid, relatively continuous anterograde and retrograde movements that span long distances to infrequent short range bidirectional movements separated by short or very long pauses [41], [42]. It is possible that NF networks form scaffolds for retaining these organelles and organizing their topography locally within axons.

Several lines of evidence from our studies indicate that Myo Va binding to NF-L regulates organelle distribution in the axon but loss of this interaction does not affect the slow or fast axonal transport cargoes ([16] and this study). However, Myo Va is implicated in the transport of neurofilaments and has been suggested to be involved in translocations of fast transport vesicular cargoes [7], [35], [43], [44]. We observed that Myo Va binds NF-L through its motor domain rather than its cargo domain (tail), making it unlikely that NFs are Myo Va cargoes. Long range Myo Va-mediated movements on NFs were also unlikely in light of our observations that NF-L deletion had no effect on the fast transport of vesicular organelles *in vivo* or *in vitro* (Rao et al., unpublished observations). Our previous studies also demonstrated that depletion of NFs and axonal Myo Va had no effect on slow axonal transport rates of NF-M or actin [16], [17]. However, the presence of NFs in the axon slows the rate of transport of Myo Va [16], presumably by the intermittent binding of Myo Va to NF-L within stationary NF networks in the axon.

An important role of NFs in maintaining the normal local three-dimensional organization of vesicular organelles in optic axons is suggested by our study. Membranous organelles were often seen closely opposed to NFs, which frequently flanked or surrounded the organelle, similar to that described for vimentin filaments and lysosomal compartments in non-neuronal cells [45]. In addition, a greater abundance of protein markers for vesicular organelles co-isolated with the cytoskeleton in optic fractions from NF-rich WT mice than from NF-L null mice. Moreover, loss of NF-L promoted the peripheral redistribution of organelles (this study) and Myo Va [16]. In further support for an organelle-anchoring role of NFs, we observed substantially reduced numbers of ER vesicles in axons of NF-L null mice despite the absence of detectable effects on fast axonal transport rates or on synthesis and export of these fast transport cargoes into axons. The absence of detectable transport changes despite decreases in organelle number in axons is not unexpected if, as observed, anchored organelles are stationary for long periods relative to their rapid movement when they are “on track”. Very small changes in the drop off rate of vesicles from the transport track into a relatively stationary pool could result in substantial reduction in the size of this pool over time without detectably altering the rate of fast transport. Because α-internexin levels are maintained in NF-L KO optic axons [46] despite marked declines in NF-M and NF-H, it is also possible that α-internexin could have compensatory effects on transport. The influence of NF-L on ER and endosome content and organization is not a non-selective effect on vesicular transport or general mechanisms regulating organelles in axons because the density of mitochondria and levels of axonal mitochondrial protein cytochrome C are increased in NF-L null optic axons (Rao et al., unpublished results). Similar increases in mitochondria and their protein markers have also been observed in desmin null mice and in cells where the vimentin cytoskeleton is disrupted [47], [48].

Bacterially expressed NF-L and Myo Va fusion proteins that are deficient in post-translational modifications such as phosphorylation and glycosylation bound each other directly without an adaptor protein in our *in vitro* binding studies. This observation implies that posttranslational modifications do not regulate the association between these two proteins. Our observation that Mg2+ enhances the Myo Va and NF-L interaction raises a possibility that changes in levels of these ions may regulate the binding activity between these two proteins. A similar mechanism has been proposed for the regulation of interaction between Myo Va and Syntaxin-1A [30].

**Figure 6:**
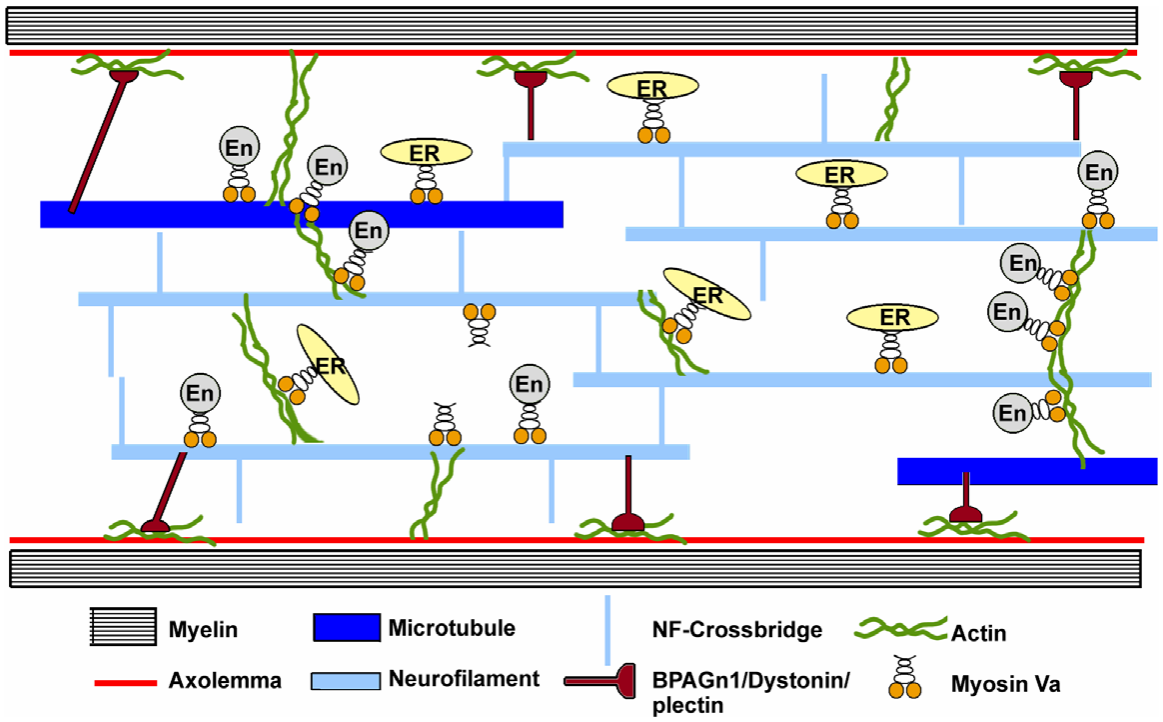
A model depicting NF-mediated anchoring and movement of organelles via Myo Va. Tethering of ER, endosomes (En), and other related vesicles to NFs are mediated by Myo Va to maintain normal abundance and distribution of these organelles in the axoplasm. The abundance of NFs in large caliber axons and their intercalation with actin and microtubule systems provides a physical continuity that may facilitate local distribution of organelles along these different filament systems via Myo Va. Loss of Myo Va-NF-L interaction in axons leads to reduced abundance of organelles as they resume transport and are ultimately turned over in the cell body.

Our studies identify the NF-L rod domain as the Myo Va binding site that forms the backbone of NFs, The abundance of NF-L subunits in the NFs (4∶2∶2∶1 stoichiometry with NF-M, α-internexin and NF-H) [17] and the putative heptad arrangement of rod domains forming the NF core represents a continuous linear array of Myo Va binding sites along axons suitable for organelle attachment through Myo Va. Moreover, as axons expand in caliber, the growth of the actin cytoskeleton does not keep pace with increases in axonal volume and NF networks. This circumstance leads to markedly lower ratios of actin filaments to NFs in large caliber axons compared to smaller ones [49]. Our immunogold data on axons showing the actin network to be discontinuous (Rao et al., unpublished observations) suggests that its networking with both microtubules and NFs might provide the continuity needed for distributing organelles radially and longitudinally and retrieving them for movement on actin or microtubules to a new location. In this regard, recent evidence suggests that myosin, kinesin, and dynein motor systems can transport the same cargo on either actin or microtubule based platforms [50]. It is also proposed that Myo Va diffuses through actin and microtubule intersections to transport cellular organelles [50], [51]. However, one study suggests that changing the track for Myo Va from actin to microtubules happens directly without diffusion [52]. A similar mechanism may be envisioned for the on- or off-loading of organelles from NFs to actin filaments or microtubules by Myo Va (Fig. 6). Although ATPase activity of Myo Va is not required for binding to NF-L, the binding of Myo Va to NF-L through its motor domain raises the possibility that Myo Va may be able to distribute cargoes along the NF.

Our studies confirm and extend our previous observations that Myo Va binds selectively not only to NF-L but also to other IF proteins of several classes, including α-internexin, GFAP, peripherin, vimentin, and desmin, but not keratin. A Blast analysis of the IF proteins identified a highly conserved (EREGLEETLRNL) sequence within the rod domain of IF proteins that bind Myo Va (NF-L, vimentin, peripherin, desmin, α-internexin, and GFAP) compared to IFs that do not bind Myo Va (NF-M, NF-H and keratin). These observations suggest that IF networks may serve as linear arrays for Myo Va-mediated organelle distribution in a variety of cell types. In this regard, dynamic rearrangements of vimentin have been shown to be important in modulating the centrifugal movements of melanosomes in B16 cells [36]. Myo Va mediated melanosome transport studies in *Xenopus* melanophores indicate that IFs significantly slow down the run lengths of the motor on microtubules. This particular study suggests that IFs hinder Myo Va dependent organelle transport in melanophores [52] and may thereby contribute to the three-dimensional positioning of these organelles in the cytoplasm.

## Supporting Information

Figure S1 **Immunoblot analyses of Myo Va and NF-L constructs.** Bacterially expressed Myo Va head (5-752, 120-kDa), neck (760-922, 60-kDa) and tail domains (899-1830, 140-kDa) Coomassie stained (A) and immunoblotted with anti-MBP antibody (B). Myosin Va head region of Myo Va was further deleted to make 41-505 (95-kDa), 41-326 (75-kDa), and 327-505 (52-kDa) constructs (C), and the neck mutants 800-881 (50-kDa), 604-881 (85-kDa), 717-799 (65-kDa) (D) were immunoblotted with MBP antibody. His-tagged tail domain deletion mutants (952-1852, 120-kDa; 952-1256, 45-kDa and 1257-1852, 62-kDa) were immunoblotted with hexa-His antibody (Fig. 1SE). (F) Purified (lanes 1, 3, 5&7), and bacterially expressed (lanes 2, 4, 6&8) Myc tagged NF-L (Coomassie stained, lanes 1&2), immunoblotted with NF-L specific (NR-4, lanes 3&4, and 21.4, lanes 5&6) and anti-Myc (9E10) antibodies (lanes 7&8). (G). Bacterial expression of Myc-tagged NF-L mutants. Lane 1: full length NF-L (1-543, 68-kDa); lane 2: C-terminal deletion mutant 1-369 (37-kDa); lane 3: 1-243 (27-kDa); lane 4: N-terminal rod domain 94-243 (20-kDa), and lane 5: 1-93 (17-kDa) were immunoblotted with anti-Myc antibody. The positions of the protein bands on all membranes are indicated with arrows.
(TIF)
