# Supplementary figures and images for: The Myosin Va Head Domain Binds to the Neurofilament-L Rod and Modulates Endoplasmic Reticulum (ER) Content and Distribution within Axons
Source: PLoS One. 2011 Feb 16;6(2):e17087. doi: 10.1371/journal.pone.0017087 (PMC3040190; doi:10.1371/journal.pone.0017087)

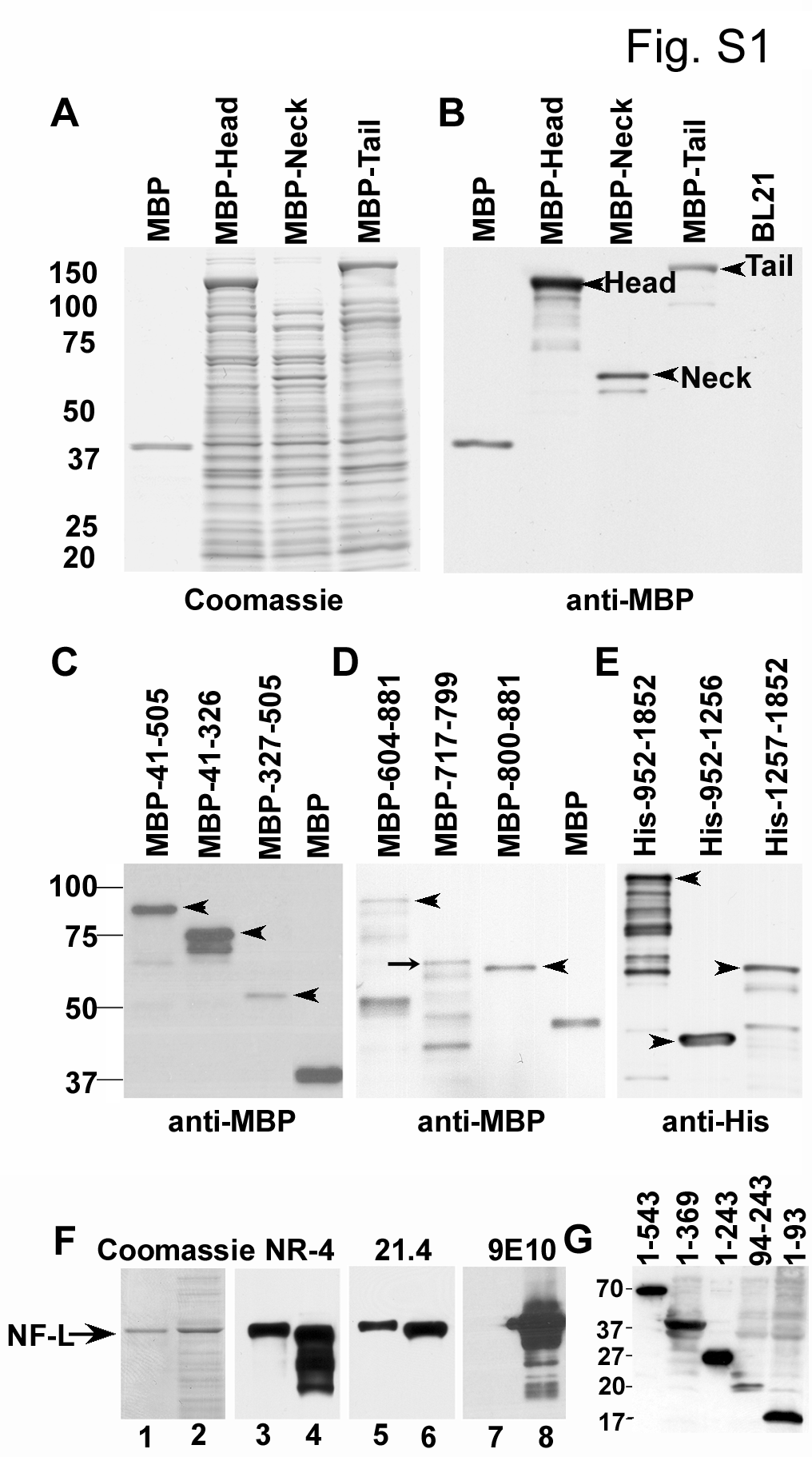

Supplement: Figure S1 — Immunoblot analyses of Myo Va and NF-L constructs. Bacterially expressed Myo Va head (5-752, 120-kDa), neck (760-922, 60-kDa) and tail domains (899-1830, 140-kDa) Coomassie stained (A) and immunoblotted with anti-MBP antibody (B). Myosin Va head region of Myo Va was further deleted to make 41-505 (95-kDa), 41-326 (75-kDa), and 327-505 (52-kDa) constructs (C), and the neck mutants 800-881 (50-kDa), 604-881 (85-kDa), 717-799 (65-kDa) (D) were immunoblotted with MBP antibody. His-tagged tail domain deletion mutants (952-1852, 120-kDa; 952-1256, 45-kDa and 1257-1852, 62-kDa) were immunoblotted with hexa-His antibody (Fig. 1SE). (F) Purified (lanes 1, 3, 5&7), and bacterially expressed (lanes 2, 4, 6&8) Myc tagged NF-L (Coomassie stained, lanes 1&2), immunoblotted with NF-L specific (NR-4, lanes 3&4, and 21.4, lanes 5&6) and anti-Myc (9E10) antibodies (lanes 7&8). (G). Bacterial expression of Myc-tagged NF-L mutants. Lane 1: full length NF-L (1-543, 68-kDa); lane 2: C-terminal deletion mutant 1-369 (37-kDa); lane 3: 1-243 (27-kDa); lane 4: N-terminal rod domain 94-243 (20-kDa), and lane 5: 1-93 (17-kDa) were immunoblotted with anti-Myc antibody. The positions of the protein bands on all membranes are indicated with arrows. (TIF) [file pone.0017087.s001.tif]
